# A Systematic Review: Is Early Fluid Restriction in Preterm Neonates Going to Prevent Bronchopulmonary Dysplasia?

**DOI:** 10.7759/cureus.50805

**Published:** 2023-12-19

**Authors:** Suresh Kumar Yadav Bollaboina, Ashok Kumar Urakurva, Saritha Kamsetti, Rakesh Kotha

**Affiliations:** 1 Pediatrics, Niloufer Hospital, Hyderabad, IND; 2 Pediatrics, Government Medical College Vikarabad, Vikarabad, IND; 3 Neonatology, Osmania Medical College, Hyderabad, IND

**Keywords:** fluid management, sodium, broncho pulmonary dysplasia, early fluid restriction, preterm neonates

## Abstract

Preterm birth causes constant challenges, with bronchopulmonary dysplasia (BPD) being a major concern. Immediately after birth, it takes time to establish feeding between the mother and the premature baby. During this time, the telological shifting of fluid from extracellular space to intracellular space will help the baby; this transition should be smooth. Both normal physiologic changes and pathophysiologic events are capable of disrupting this delicate fluid shifting that occurs in very low-birth-weight infants during the first week of life. The immaturity of the renal system and evaporative losses complicate this process. This lack of fluid displacement can be associated with an increased amount of water in the lungs and reduced lung compliance. This can lead to the need for more ventilatory support and a higher oxygen requirement, which, in turn, leads to lung damage. The fluid restriction is also associated with complications such as severe dehydration, intracranial hemorrhage, and bilirubin toxicity. However, the administration of large amounts of fluid and salt is associated with an increased incidence of patent ductus arteriosus, BPD, necrotizing enterocolitis, and intraventricular hemorrhage. There were studies conducted in both the pre-surfactant and surfactant eras that were inconclusive regarding fluid restriction in BPD. We only included very recent studies. This systematic review attempts to summarize the current evidence, focusing on the efficacy and safety of early fluid management in preterm infants. This reduces the risk of BPD and improves outcomes for premature infants. As we know, intact survival is very important. Our review supported the early fluid restriction.

## Introduction and background

Bronchopulmonary dysplasia (BPD) is a common and serious disease in preterm neonates [1]. The reported global incidence range of BPD was 10-89% [2]. Management practices and the genetic diversity of the populations studied can explain this wide range. In addition to prematurity, low birth weight (LBW), and respiratory distress syndrome (RDS), several neonatal factors have been implicated in the pathogenesis of BPD. These include patent ductus arteriosus (PDA), sepsis, increased hydration, increased ventilator support, and supplemental oxygen. Antenatal corticosteroid use and permissive hypercapnia, on the other hand, have been associated with a lower incidence of BPD [3-5].

The relationship between fluid administration and the development of BPD in preterm neonates is a subject of ongoing research [6]. While fluid management is critical in the care of neonates, the idea of early fluid restriction in preventing BPD remains inconclusive [7]. However, multiple studies have indicated that restrictive fluid strategies aiming to avoid fluid overload reduce the risk of BPD. The rationale for restricting fluid intake is that abnormal fluid intake causes respiratory distress, makes preterm neonates use mechanical ventilation, and leads to the development of BPD. High fluid input increases the risk of a PDA, a major cause of BPD. [8,9].

In newborns, there tends to be an inverse relationship between extracellular fluid and gestational age. Inappropriately high fluid and sodium intake in the early days of a preterm neonate may prevent the extracellular fluid from contracting physiologically, which may lead to BPD [10-13]. Any intervention aimed at one system will have an impact on other systems as well. Fluid management affects nutrition and electrolytes and has cardiopulmonary consequences; ventilator practices will also affect cardiovascular function. In this review, we focused only on the effects of early fluids on BPD.

## Review

Objective

The primary aim of conducting this thorough and rigorous systematic review is to offer a valuable and comprehensive understanding of the association between early fluid restriction during the early stages of life and the development of BPD in preterm neonates.

Methodology

Study Selection Criteria

We focused on preterm neonates as the study population, fluid restriction as the intervention, and BPD-free preterm neonates as the outcome. The inclusion criterion encompassed studies reporting on early fluid restriction in preventing BPD in preterm neonates. Studies that did not report on fluid restriction in preventing BPD in preterm neonates or studies with methodologies inadequacies, insufficient information, or insufficient interventions were excluded (Table 1). Through this selection criterion, we managed to identify studies that reliably established the efficacy of fluid restriction among preterm neonates in preventing the development of BPD. The study was registered under PROSPERO with ID 487318.

**Table 1:** Inclusion and Exclusion Criteria

Inclusion Criteria	Exclusion Criteria
Cohort studies with findings on the impact of fluid restriction on BPD.	Opinion or commentary articles that provide insights about the role of early fluid restriction in preterm neonates in preventing BPD.
Cohort studies that have investigated the relationship between fluid intake management in preterm neonates and the development of BPD.	Studies focusing on early fluid restriction in preterm neonates without linking it to BPD. Studies not related to early fluid restriction in preterm neonates in preventing BPD.
Case control studies that have investigated interventions related to fluid restriction in preventing the development of BPD in preterm neonates.	Conference papers or abstracts on early fluid restriction in preterm neonates in preventing BPD that lack comprehensive data and results.
Studies that have investigated interventions related to fluid restriction in preventing the development of BPD in preterm neonates within the last decade.	Studies that have not investigated interventions related to fluid restriction in preventing the development of BPD in preterm neonates in the last decade.
Randomized control studies that focused on interventions that prevent the development of BPD in preterm neonates.	Studies that do not focus on preterm neonates.

Search Strategy

A search strategy involves looking for articles that address the research topic. For this study, the search strategy followed the PICO (population, intervention, comparison, and outcome) framework, which provided a guide for leading relevant articles that addressed the research topic (Table 2). Using the PICO approach, elements of the research topic that form the PICO framework were established. We used Boolean operators and searched across databases, particularly Scopus, EMBASE, and PubMed. Keywords underlying the PICO framework combined using Boolean operators such as “and” include “early fluid restriction,” “preterm neonates,” and “bronchopulmonary dysplasia."

**Table 2:** PICO Framework PICO: Population, intervention, comparison, and outcome

PICO Framework	
Population	Neonates
Intervention	Fluid restriction in early days
Comparison	Standard fluid protocol
Outcome	Broncho pulmonary dysplasia
Study	Observational and Randomization studies

In addition, alternative terms were used to identify articles that could help address the research topic. For example, sodium restriction was used in place of fluid restriction to identify articles that could contribute to addressing the research topic (Table 3).

**Table 3:** Search Strategy MeSH: Medical Subject Headings

S.NO. & Search	Search Strategy
# 1. Search	Early fluid restriction in preterm neonates will prevent the broncho pulmonary dysplasia [Mesh terms]
#2. Keywords search	preterm neonates, early fluid restriction, broncho pulmonary dysplasia, sodium, fluid management [Title/Abstract]
#3. Keyword	Early fluid restriction in preterm neonates and pulmonary dysplasia [truncation]
#4 Keyword	Interventions related to fluid restriction in preventing the development of BPD in preterm neonates [Alternatives]
#5. Keyword search	Fluid status in preterm neonatal and broncho pulmonary dysplasia [Truncation/alternatives]

PRISMA Flow Diagram

**Figure 1:**
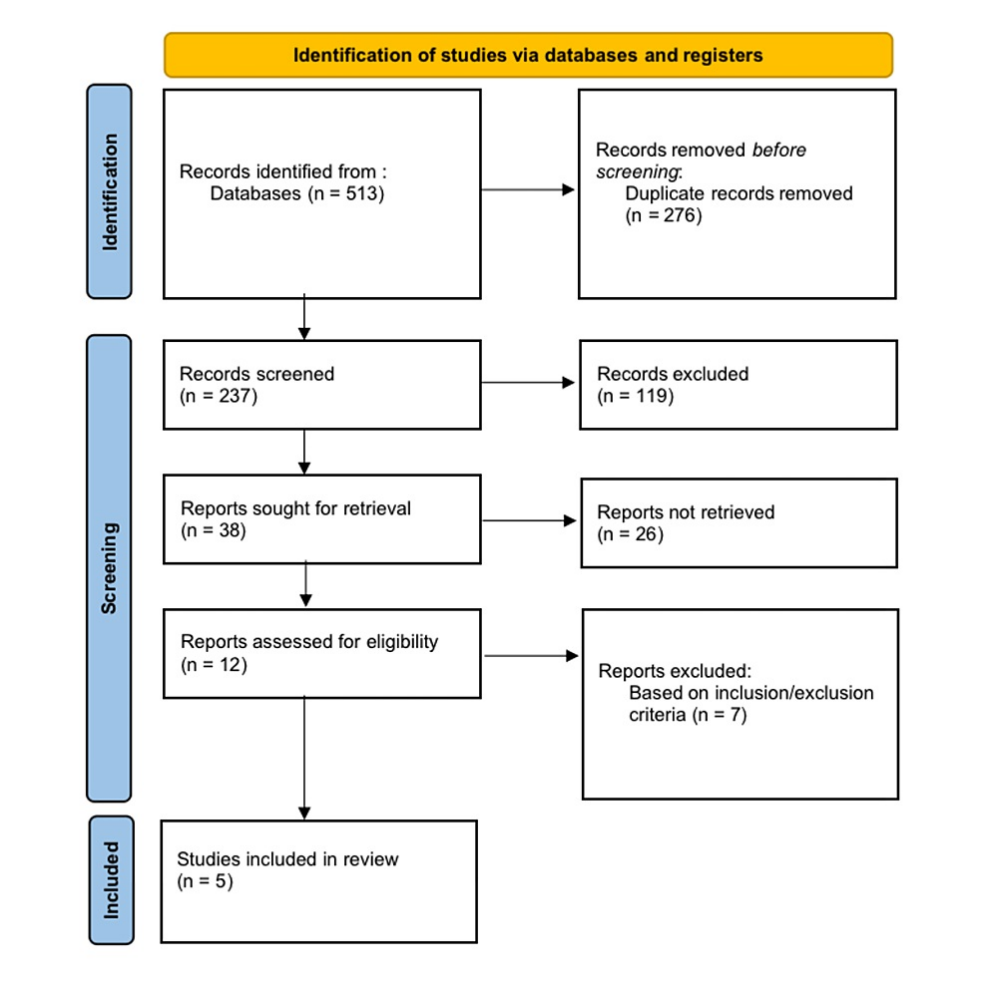
PRISMA Flow Diagram for the Selection of the Articles

The selection process for identifying quality articles is represented in the PRISMA flow diagram. The PRISMA flow diagram provided a reflection of the guide for identifying quality articles. The key elements of the PRISMA flow diagram for this study included article identification and screening, establishing eligibility, and weighing against the inclusion/exclusion criteria (Figure 1). The key elements of the PRISMA flowchart for this study included identifying and screening articles, determining eligibility, and balancing against the inclusion/exclusion criteria (Figure 1).

The EndNote tool (Clarivate, London, UK) was used to search all these sources for suitable articles relevant to our topic. The articles found were manually reviewed to exclude duplicates. The selection of relevant publications addressing the topic of early fluid restriction in BPD was a further step in the screening process. The content of the topics, the abstracts, the main results, conclusions, and overall information of the publications were examined to find the correct ones. Through the search strategy, several possible publications (513), with links to the research topic, were found. Of the many articles found, only five met the threshold to be included in the systematic literature review. The articles that were initially identified in the peer-reviewed articles were included and considered for the systematic literature review.

Types of Studies Used

This systematic evaluation focused on randomized control trial studies, retrospective cohort studies, and retrospective case-control studies. Randomized and retrospective cohort studies were suitable for this systematic review because they provide valuable insights into relationships between exposures and outcomes. Additionally, randomized control trials and cohort studies are observational in design, making them suitable for tracking and justifying the impact of interventions on health concerns or problems.

Equally, this systematic evaluation focused on retrospective case-control studies. Retrospective case-control studies were considered because they provide an association between a disease or health condition and potential risk factors or exposures. The assessment of the quality of studies for this review was done using the Newcastle-Ottawa scale. According to this tool, five studies were of good quality (Table 4).

**Table 4:** Quality Ratings of the Selected Articles Based on the Newcastle-Ottawa Scale

SN	Study	Selection	Comparability	Outcome
1	Li et al. (2022) [7]	***	**	***
2	Oh et al. (2005) [16]	***	**	***
3	Uberos et al. (2020) [14]	***	**	***
4	Cunha et al. (2022) [17]	***	**	***
5	Soullane et al. (2021) [15]	***	**	***

Li et al. included 157 babies in their case-control study, which was analyzed retrospectively. ROC curves were used to determine the early seven-day cumulative fluid load on BPD. The study discovered that a seven-day cumulative fluid overload threshold of 36.2% is the most accurate predictor for the development of BPD in preterm neonates [7].

Uberos et al., in their cohort study, included 389 babies, of whom 159 developed BDP. They found that infants with a lower intake of energy and lipids and a higher intake of fluids in the first week of life were more susceptible to BPD. However, Uberos et al.'s study primarily focused on the association between energy intake and BPD [14].

Soullane et al.'s retrospective cohort study included 191 preterm infants born between 23 and 28 weeks of gestation. The data was taken from the local Canadian Neonatal Network (CNN) database. In this study, the multivariable logistic regression analysis revealed that higher cumulative fluid balance was linked to increased odds of death (BPD). The absolute odds ratio (AOR) was 1.56, and the 95% confidence interval limit was (CI 1.11-2.25) [15].

Similarly, Oh et al. highlighted that higher fluid intake and a lack of weight loss were significantly associated with a greater risk of death or BPD (p < 0.001 and 0.006, respectively [16]. A major strength of the study is the large sample size; he included 1,382 infants. The researchers analyzed a randomized controlled trial about the role of parenteral glutamine supplementation in late-onset sepsis in ELBW infants. This study was done during the surfactant era [17].

A prospective cohort study conducted by Cunha et al. showed that a fluid volume of more than 131 mL/kg/day on day seven was associated with an increased risk of BPD. The relative risk (RR) was 2.09, and the 95% CI was 1.14-3.85 [18]. The major limitation of this study is that it only included 68 ventilated babies.

It was designed to assess the quality of non-randomized studies. This scale developed a "star system" in which it evaluated three general aspects: selection, comparability, and outcome. We chose this simple and practical tool for the quality assessment of non-randomized studies for our systematic reviews.

Discussion

There exist two distinct categories of BPD, namely, old BPD and newer BPD. Old BPD typically manifests itself in the pre-surfactant era and is commonly observed in babies with a gestational age of over 32 weeks. This prevalence can be attributed to the lower survival rates of infants born at lower gestational ages during that period. On the other hand, newer BPD arises as a result of various risk factors associated with the mother, fetus, and perinatal conditions. The occurrence of injury at an early stage leads to a developmental abnormality in the septation of the alveoli, making it more challenging to treat. Interestingly, studies investigating the impact of early fluid restriction on both types of BPD have yielded inconclusive results [19].

The purpose of this systematic review was to determine whether early fluid restriction in preterm neonates prevents BPD. Multiple studies on early fluid restriction in preterm neonates and BPD have been conducted [20]. However, discourses regarding the efficacy of early fluid restriction in preterm neonates in preventing BPD are ongoing. This study is one of the current systematic reviews that brings together evidence from well-structured and well-conducted cohort and case-control studies. This study provides reliable evidence that early fluid restriction in preterm infants prevents BPD [4].

The permeability of the capillaries will be higher during the first week, particularly in premature babies. The excess fluid is more likely to leak into the interstitial space of the lung. Therefore, the risk of BPD will be higher in babies who receive more fluids in the first few days of life. However, fluids, especially colloids, administered to sick premature babies after week one are also a risk factor for BPD, as capillary permeability increases in septic babies. Even the smallest babies may require more than 200 mL/kg/day to prevent dehydration and hypernatremia; this is a major concern. The level of care should also be considered when prescribing fluids, whether the baby is in an incubator or a warmer environment.

All included studies reported that fluid restriction in the early days will decrease the risk of BPD [7,14-17]. Oh et al. reported that appropriate fluid regimens for preterm neonates can prevent BPD [16]. Wadhawan et al., in their retrospective analysis, mentioned that early postnatal weight loss is associated with a decreased risk of BPD (47.2 vs. 64%, p < 0.001) [20].

Rocha et al.'s investigation into the association between fluid balance during the first 10 days of life and BPD showed that higher fluid intake during the first 10 days of life was associated with an increased risk of BPD [1]. Many researchers have conducted numerous studies regarding blood product transfusions in the early days and the risk of BPD. However, the majority of studies do not conclude whether excess volume or inflammatory damage from blood products is a risk factor for the development of BPD [21,22].

In 1990, Van Marter et al. noticed that those with BPD received greater total fluid per kilogram per day during the first four days of life [23]. Hartnoll et al. conducted a randomized trial in the surfactant era regarding early and late supplementation of sodium. They estimated the pulmonary arterial pressure (PAP) in both groups as the ratio of time to peak velocity to right ventricular ejection time, corrected for heart rate (TPV: RVET(c)). They found that PAP did not decrease until day 14 in the group that received early sodium supplementation [24].

On the other hand, some studies have established a lack of a strong correlation between early fluid restriction in preterm neonates and BPD [25]. Koo et al. found that there is no significant association between early fluid restriction and BPD [26]. Guimaraes et al. established that low birth weights and hyaline membrane disease were more significant risk factors for BPD. This suggests that the likelihood of early fluid restriction in preventing BPD is low compared to other interventions [27].

Investigations have also been conducted on the association between sodium intake in preterm neonates and BPD. Barrington reported that restricted sodium intake within the first 72 hours of age was linked to a reduced risk factor for BPD. In his review, Barrington reported a possible reduction in BPD (RR 0.76, 95% CI: 0.56, 1.04), whereas restricting free-water intake by itself has little or no effect [28]. However, there are many limitations in this study, including more heterogenicity, a smaller number of babies (274), and very few extremely premature babies. Costarino et al. reported that avoiding sodium supplements in the first week of life was linked to a reduced risk of BPD, but they were unable to conclude the role of sodium restriction [29]. Kavvadia et al. found that fluid restriction during perinatal periods does not reduce CLD in ventilated, very low-birth-weight infants, but colloid infusion increases oxygen dependency duration [30]. Niwas et al. concluded that there was no difference in the outcome of BPD in the fluid-restricted group, but he mentioned many limitations, such as the retrospective nature, including a lack of data on serum electrolytes and urine output, and a small sample size [31].

Early studies reported that sodium plays a more crucial role than free fluid in preventing BPD. Normal physiologic weight loss is deemed adequate and sufficient to prevent BPD, although, in certain studies, there are some important limitations noted, as mentioned above. Most studies, however, concluded that excessive excess fluid intake in the first few days poses a significant and major risk factor for BPD. In our review, rigorous quality assessment ensured the reliability of the identified studies. The identified studies were reliable, supporting the notion, and we concluded that early fluid restriction is pivotal in BPD prevention.

## Conclusions

This systematic review aimed to gather dependable evidence on the efficacy of early fluid restriction in preventing BPD in preterm infants. The identified studies consistently support the idea that early fluid restriction is effective in averting BPD. Advocating sodium restriction in the early days, coupled with allowing physiological fluid loss, is recommended. While some older studies contested the role of fluid restriction, probably due to diminished use of antenatal steroids and surfactants, we stress the need for more randomized, multicenter trials in this grey area where precise treatment remains elusive.
